# Decellularisation and Characterisation of Porcine Pleura as Bioscaffolds in Tissue Engineering

**DOI:** 10.1155/2024/9940673

**Published:** 2024-07-08

**Authors:** Thirapurasundari Vikranth, Tina Dale, Nicholas R. Forsyth

**Affiliations:** School of Pharmacy and Bioengineering Guy Hilton Research Centre Keele University, Keele, UK

## Abstract

Persistent air leaks caused by thoracic surgery, physical trauma, or spontaneous pneumothoraces are a cause of patient morbidity with need for extended chest tube durations and surgical interventions. Current treatment measures involve mechanical closure of air leaks in the compromised pleura. Organ and membrane decellularisation offers a broad range of biomimetic scaffolds of allogeneic and xenogeneic origins, exhibiting innate tissue-specific characteristics. We explored a physicochemical method for decellularising porcine pleural membranes (PPM) as potential tissue-engineered surrogates for lung tissue repair. Decellularised PPM (dPPM) was characterised with histology, quantitative assays, mechanical testing, and sterility evaluation. Cytotoxicity and recellularisation assays assessed biocompatibility of decellularised PPM (dPPM). Haematoxylin and Eosin (H&E) staining showed an evident reduction in stained nuclei in the dPPM, confirmed with nuclear staining and analysis (^*∗∗∗∗*^*p* < 0.0001). Sulphated glycosaminoglycans (sGAG) and collagen histology demonstrated minimal disruption to the gross structural assembly of core extracellular matrix (ECM) in dPPM. Confocal imaging demonstrated realignment of ECM fibres in dPPM against native control. Quantitative analysis defined a significant change in the angular distribution (^*∗∗∗∗*^*p* < 0.0001) and coherence (^*∗∗∗*^*p* < 0.001) of fibre orientations in dPPM versus native ECM. DNA quantification indicated ≥85% reduction in native nuclear dsDNA in dPPM (^*∗∗*^*p* < 0.01). Collagen and sGAG quantification indicated reductions of both (^*∗∗*^*p* < 0.01). dPPM displayed increased membrane thickness (^*∗∗∗*^*p* < 0.001). However, Young's modulus (459.67 ± 10.36 kPa) and ultimate tensile strength (4036.22 ± 155.1 kPa) of dPPM were comparable with those of native controls at (465.82 ± 10.51 kPa) and (3912.9 ± 247.42 kPa), respectively. *In vitro* cytotoxicity and scaffold biocompatibility assays demonstrated robust human mesothelial cell line (MeT-5A) attachment and viability. DNA quantification in reseeded dPPM with MeT-5A cells exhibited significant increase in DNA content at day 7 (^*∗∗*^*p* < 0.01) and day 15 (^*∗∗∗∗*^*p* < 0.0001) against unseeded dPPM. Here, we define a decellularisation protocol for porcine pleura that represents a step forward in their potential tissue engineering applications as bioscaffolds.

## 1. Introduction

Advances in tissue engineering (TE) and biomaterial sciences have revolutionised the scope of biomimetic transplant fabrication, capable of replacing and restoring damaged tissue structure and function. Whole organ and membrane decellularisation approaches have produced biological scaffolds that exhibit the structural and functional attributes of native ECM. With ECM composition and architecture being relatively conserved amongst species [1, 2], the options of using accessible, economically viable, and biocompatible xenogeneic scaffolds have considerably expanded with decellularisation. Decellularised porcine small intestinal submucosa (SIS) [3] and urinary bladder matrix (UBM) [4], derived by mechanical delamination of the tunics, have shown excellent retention of native ECM histoarchitecture and biomechanics, gaining clinical applications in genitourinary reconstruction [5–7] and soft tissue augmentation [8–11]. Decellularised porcine dermis processed as freeze-dried sheets, has been used to promote reepithelialisation in full- and partialthickness wounds and burns [12, 13]. Lung tissue decellularisation has also gained considerable research interest with the growing need for donor grafts, as lung transplantation remains the only definitive treatment in end-stage lung disease [14–16]. However, the regionally complex and specific cellularity and extracellular matrix (ECM) distribution in lungs challenges the standardisation of a decellularisation protocol applicable for the entire lung tissue. Along with efforts in deriving acellular whole lungs, recent studies have narrowed down approaches to specific regions in lung tissue based on intended applications as airway or vascular grafts or in fibrotic and airway disease models [17–19].

Air leaks that remain unresolved for longer than 5 days are clinically defined as persistent air leaks (PAL). At postsurgical incidence of 23% following tumour resections, PAL pose significant morbidity concerns associated with extended chest tube durations, secondary pulmonary complications, and prolonged hospital stays [20, 21]. Current clinical management involve intraoperative closures such as buttressing staple lines, pleurodesis, and use of biological and composite sealants [22, 23]. Fibrin, a routinely used natural sealant known for its biocompatibility, lacks mechanical strength in sustaining long-term closure of surgical incisions. Retrospective studies in air leak management are yet to provide compelling evidence to suggest a superior technique in preventing PAL. Most studies report a marginal decrease in the incidence of PAL with no significant reduction in chest tube durations or hospital admissions [21, 24–27]. In addition, the use of sclerosing agents and composite sealants has been associated with unfavourable immune reactions in patients.

Pleurae that form the thin serosal lining of the lungs and the thoracic cavity are defined by a superficial mesothelial cell monolayer resting on a thin basement membrane and an underlying loose connective tissue matrix rich in collagen and elastin [28, 29]. Apart from providing a protective low-friction barrier for smooth movement of lungs within the thoracic cavity, pleural ECM plays a dual mechanical role in limiting lung distensibility and providing elastic recoil during breathing [30]. The inherent immunomodulatory and inflammatory repertoire of secretory factors released in response to tissue injury enables pleura for spontaneous repair and wound healing [31, 32]. However, this regenerative potential is limited to the extent and severity of pleural injury and disease [33].

We aimed to develop a reproducible decellularisation protocol for porcine pleura and assess protocol efficiency in adequate cell removal and preservation of innate structural and functional characteristics of pleural ECM. Deriving mechanically sound and biocompatible acellular pleural membranes could potentially offer tissue-specific applications as sealant grafts in air leak management and pleural repair.

## 2. Materials and Methods

### 2.1. Decellularisation of PPM

Fresh pig lungs sourced from a local abattoir were used for pleural membrane excisions. Excision was carried out in a class I dissection hood with lungs placed on crushed ice. PPM were peeled gently and transferred to Petri-dishes containing chilled phosphate-buffered saline (PBS, 1x without calcium and magnesium, pH 7.4 ± 0.1, Corning®, USA). Excised membranes were rinsed thoroughly in deionised water to remove blood and cell debris.

**Figure 1:**
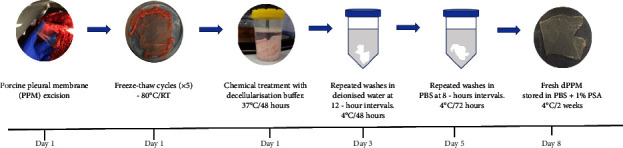
Schematic representing protocol for porcine pleural membrane (PPM) decellularisation.

As in Figure 1, PPM disruption and cell lysis was initiated with 5x freeze-thaw cycles of snap freezing at −80°C for 15–20 min followed by thawing at room temperature for 25–40 min. Repeated washing with deionised water ensured removal of extraneous matter and cell debris. Samples were next immersed in a decellularisation buffer, (1% (v/v)) Triton-X 100, 0.5% sodium deoxycholate in 10 mM Tris buffer pH 7.6 (both Sigma-Aldrich) and 30 *µ*g/mL DNase I solution (Invitrogen™, USA), for 48 hours at 37°C under mild agitation. Treated samples were refrigerated and washed 6x with deionised water at 8 hour intervals and then 6x with PBS at 12 hour intervals. dPPM were stored at 4°C in PBS containing 1x penicillin/streptomycin/amphotericin B solution (100X PSA containing 10,000 units potassium penicillin/mL, 10,000 *µ*g streptomycin sulphate/mL and 25 *µ*g amphotericin B/ml in 0.85% saline, Lonza, Switzerland) for two weeks before being transferred to −80°C, for long term preservation. Three biological replicates with five technical repeats for native and dPPM samples were studied.

### 2.2. Histology

dPPM was stained with H&E (hematoxylin solution, Gill no. 2, Sigma-Aldrich, aqueous Eosin Y, Thermo Scientific) to assess cellular content and histoarchitecture. Picrosirius red (PSR) (Sigma-Aldrich) staining was used to visualise collagen distribution and orientation while Alcian blue (1% Alcian blue in 3% acetic acid, Thermo Fisher Scientific, MA, USA) staining was used to qualitatively examine sGAG content. Elastin fibres were examined using standard IHC protocol for Verhoeff – Van Gieson (VVG) staining. Stained samples were dehydrated using a graded alcohol series and mounted with DPX (360294H, Analar) mounting media. Phase contrast and bright field images were acquired at 20x magnification (EVOS digital imaging systems, AMG Washington).

### 2.3. Confocal Imaging and Analysis

Fresh native and dPPM samples were imaged under a laser scanning confocal microscope (Olympus Fluoview FV1200, Japan) at 20x magnification. Autofluorescence of ECM fibres was detected in the samples when excited with a 488 nm multiline Argon laser. Image acquisition and control was carried out using the integrated FV10–ASW software. The 2D images acquired, were in ImageJ compatible OIB (Olympus image binary) formats with a resolution of 1024 × 1024 pixels (physical size = 636 microns × 636 microns, 16-bit).

OrientationJ plugin for ImageJ/Fiji was used for image analysis. A 10-pixel Gaussian analysis window with an estimated cubic spline gradient was specified for the distribution and measurement modes in OrientationJ. Structure tensors computed by sliding the Gaussian analysis window across the image, was used for determining local orientations of ECM fibres. Orientations ranging from −90° to +90° from the horizontal, were represented as colour-coded orientation maps and histograms of the distribution of fibre orientations in native and dPPM, using the OrientationJ distribution mode. Measurement mode of OrientationJ quantified differences in coherency and orientation of ECM fibres between native ECM and dPPM. Each image was analysed with eight regions of interest (ROI) selected using the rectangular select tool in ImageJ. Best fitting ellipses created for each ROI defined features of the gradient structure tensors with respect to fibre orientations and coherency. Angular distribution of fibres and the degree, to which these features were oriented in each ROI, was tabulated as orientation and coherency. Orientation values were represented in degrees and coherency values as an index between 0 and 1. Coherency of 1 indicated a single dominant fibre orientation in the ROI while 0 indicated fibre isotropy in orientation.

### 2.4. Nuclear Labelling and DNA Quantification

Nuclear DNA integrity and gross cell morphology in dPPM were assessed with 4′, 6′-diamidino-2-phenyl indole (DAPI, Sigma-Aldrich) staining and fluorescence microscopy (Nikon Eclipse Ti, Japan). Nuclear quantitation was performed using the analysis plugin of ImageJ (Rasband, W.S., ImageJ, U.S. National Institutes of Health, USA).

Native PPM and dPPM samples were desiccated in a hot air oven overnight to a constant weight (approximately 25 mg. dry weight) prior to DNA quantification. Samples were treated overnight with proteinase K (1.25 mg/mL, Sigma-Aldrich) at 55°C, to obtain clear membrane digests. Residual dsDNA in dPPM and native digests was quantified using the Quant-iT™ PicoGreen™ dsDNA assay kit (Thermo Fisher Scientific, USA) following manufacturer's instructions. Samples were read at 480 nm and 520 nm excitation and emission wavelengths respectively, on a fluorescence microplate reader (BioTek Synergy 2 plate reader, USA). dsDNA content was calculated using the standard calibration curve, following manufacturer's instructions, and expressed as *µ*g/mg. dry weight of tissue.

### 2.5. sGAG Quantification

Samples were first dried and digested as described above. Quantification of sGAG content was established using the 1,9-dimethyl methylene blue assay (DMMB, Abcam, UK) following manufacturer's instructions. Digested samples (50 *µ*L) were aliquoted in a 96-well microplate. 200 *µ*L of the DMMB solution (16 mg DMMB, 0.76 g glycine, 0.595 g NaCl, 23.75 mL of 0.1 M HCl, and distilled water to 250 mL) were added to each well using an automated dispenser. Sample absorbance was immediately measured at 530 nm on a Synergy 2 microplate reader. sGAG content expressed as *µ*g/mg. dry weight of tissue, was determined against a standard calibration curve, following manufacturer's instructions.

### 2.6. Collagen Quantification

Native and dPPM samples were prepared using acid-pepsin extraction guidelines from the manufacturer (Sircol™, insoluble collagen assay S2000, Biocolor Ltd.). Samples were then dried and treated overnight with the acid-pepsin solution (0.1 mg/mL pepsin in 0.5 M acetic acid, Sigma-Aldrich) at 4°C on a mechanical shaker. Digested samples were centrifuged at 3000 g for 10 minutes to obtain residual tissue for the insoluble Sircol-binding collagen assay following manufacturer's guidelines. Sample absorbance was measured at 556 nm on a Synergy 2 microplate reader. Insoluble or native collagen content was determined against a standard calibration curve and expressed as *µ*g/mg. dry weight of tissue.

### 2.7. Elastin Quantification

Elastin content in native and dPPM tissue samples was determined using Fastin colorimetric assay following manufacturer's guidelines (Fastin™, elastin assay kit, Biocolor Ltd.). Samples were treated with 0.25 M Oxalic acid at 100°C for 60 minutes. Following which, samples were centrifuged at 13000 g for 10 minutes to acquire the first supernatant extract. Residual tissues were subjected to at least three similar cycles before analysis. Final extracts were processed following manufacturer's guidelines for the formation of an elastin-dye complex. Sample absorbance was measured at 513 nm on a Synergy 2 microplate reader. Elastin content was determined against a standard calibration curve and expressed as *µ*g/mg. dry weight of tissue.

### 2.8. Biomechanical Studies

Membrane thickness of native and dPPM samples were measured using an optical tensiometer (Attention, Biolin Scientific, Manchester, UK) and ImageJ software. Fresh dPPM and native samples cut into 4 cm × 1 cm sections, were clamped between the upper (mobile) and lower (stationary) grips of a benchtop tensile testing apparatus (Bose ELF 3200, Minnetonka, USA). Samples were subjected to uniaxial tension to failure, at a load of 22 N and relative axial displacement of 10 mm at 0.1 mm/sec. Initial length (*L*_0_) was kept consistent at 1 mm using a digital calliper. WinTest®7 software was used for data acquisition. Resulting stress-strain curves were used to calculate Young's modulus and ultimate tensile strength of samples.

### 2.9. Sterility Evaluation

For sterilisation with UV, dPPM samples (*n* = 5) were irradiated using the preprogrammed sterilisation setting (maximum wavelength of 253.7 nm, 90 seconds exposure) on the GS Gene Linker UV chamber (Bio-Rad laboratories, California, USA). Samples placed in Petri dishes received three repeats of irradiation for top and bottom surfaces of the dish. Alternatively, dPPM samples (*n* = 5 for each treatment) were treated with either 10x PSA, 90% industrial methylated spirits (IMS), or 0.1% peracetic acid (PAA) in 4% aq. ethanol for 30 min. After treatment, samples were rinsed in sterile PBS and transferred to 24-well plates. Dulbecco's modified eagles medium (DMEM, 4.5 g/L glucose, sodium pyruvate without L-glutamine, Corning), containing 10% fetal bovine serum (FBS, Biosera), was added to each well and the plates were incubated under standard conditions (37°C, 5% CO_2_, and 95% relative humidity). Sample wells were assessed visually at days 0, 3, and 7 for infection, to determine sterilisation efficiency.

### 2.10. *In Vitro* Cytotoxicity Assay

Human mesothelial cells (MeT-5A, ATCC CRL-9444™, Virginia, USA) were cultured in cFAD medium [34], composed of DMEM and Hams F-12 (Lonza), (3 : 1, v/v), supplemented with 10% FBS, 1% nonessential amino acids (Lonza), 2 mM L-glutamine (200 mM, Lonza), 1% sodium pyruvate (100 mM, Lonza), 1% PSA, 24 *µ*g/mL Adenine (Sigma-Aldrich), 0.4 *µ*g/mL hydrocortisone (Sigma-Aldrich), 0.13 *µ*g/mL triiodothyronine (Sigma-Aldrich), 10 ng/mL epidermal growth factor (EGF, Sigma-Aldrich), 5 *µ*g/mL transferrin (Sigma-Aldrich), 5 *µ*g/mL insulin (Sigma-Aldrich). Cells were seeded at 0.1 × 10^5^ cells/mL on collagen coated 24-well plates and incubated under standard culture conditions. After 48-hours, 5 mm × 5 mm sections of sterilised dPPM were placed in the wells. Cells incubated in cFAD with no contact with dPPM and cells treated with 70% methanol for 30 min served as controls for live-dead staining, respectively. Fluorescence viability assay using the live/dead staining kit (Invitrogen™ L3224) was carried out following manufacturer's instructions. Sample wells were imaged using dual channel filters, FITC for live cells and Texas Red for dead cells. Cell viability was determined using Trypan blue (Lonza) exclusion assay to estimate viable cell counts at specific timepoints from day 0–day 5 of incubation.

### 2.11. Scaffold Biocompatibility

Sterilised dPPM cut into 5 mm × 5 mm sections, were mounted on sterile 24-well CellCrown™ inserts (Scaffdex Oy, Sigma-Aldrich, USA) and placed in individual wells of a 24-well plate. MeT-5A cells were labelled with a live cell tracking dye (Vybrant® DiO (1 mM), Invitrogen™ V22889) following manufacturer's recommended protocol and seeded at a density of 0.3 × 10^5^ cells/cm^2^, on sterile dPPM inserts. Sample wells were incubated under standard cell culture conditions and imaged periodically. Labelled MeT-5A cells seeded on tissue culture plastic (TCP) and sterile dPPM inserts served as positive and negative controls, respectively.

### 2.12. Statistical Analysis

Statistical analysis was performed using GraphPad Prism version 9.0 software (GraphPad Software Inc., San Diego, CA). Data were represented as mean ± standard deviation (SD) at 95% confidence intervals for each study. Results for dPPM and native samples from mechanical testing and quantitative assays were compared using an unpaired Students *t*-test. DNA quantification results were analysed using ordinary one-way ANOVA test to determine statistical significance. Probability values of ^*∗*^*p* < 0.05, ^*∗∗*^*p* < 0.01, ^*∗∗∗*^*p* < 0.001, ^*∗∗∗∗*^*p* < 0.0001 were considered statistically significant.

## 3. Results

### 3.1. PPM Decellularisation and Histology

**Figure 2:**
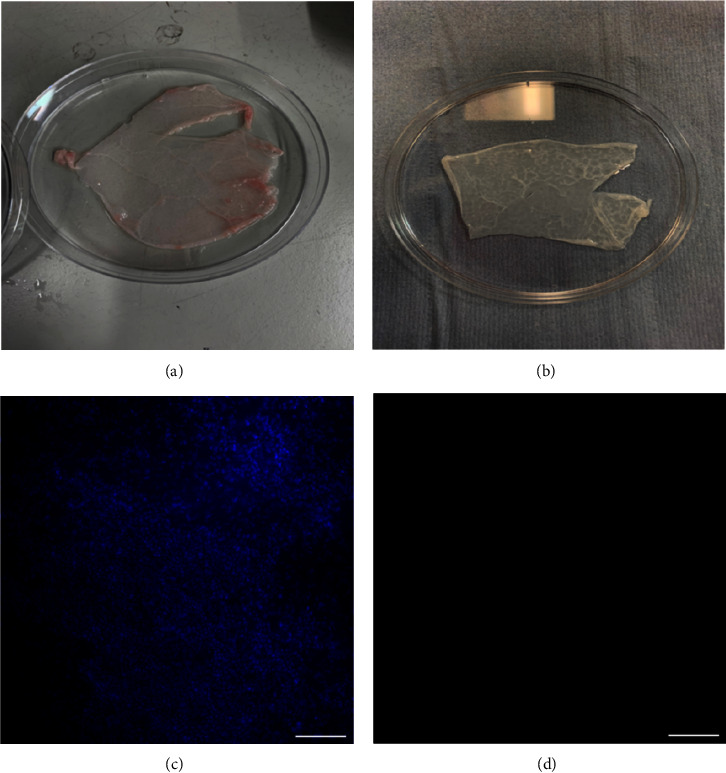
Characterisation of native and decellularised PPM. (a, b) Represent macroscopic changes to the native PPM (a) following decellularisation (b). (c, d) Represent the visible reduction in DAPI stained nuclei in dPPM (d) against native PPM (c). Scale bar = 200 *µ*m.

Macroscopically, native PPM (Figure 2(a)) appeared white and opaque following decellularisation (Figure 2(b)). Fluorescence imaging of DAPI-stained nuclei in native (Figure 2(c)) and dPPM (Figure 2(d)) showed a marked reduction in cellularity in dPPM.

**Figure 3:**
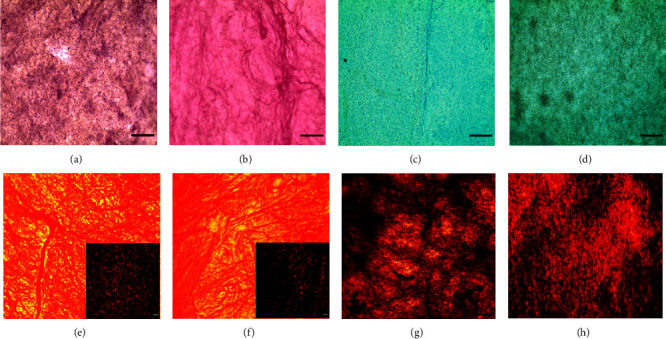
Histological features of native and decellularised PPM. (a, b) Hematoxylin and Eosin (H&E) of native (a) and dPPM (b). (c, d) Alcian blue staining in native PPM (c) and dPPM (d). (e, f) Picrosirius red staining in native ((e) inset under cross-polarised light) and dPPM ((f) inset under cross-polarised light). (g, h) Verhoeff–Van Gieson (VVG) staining in native (g) and dPPM (h). Scale = 100 *µ*m.

Histological features in dPPM demonstrated changes relative to the microanatomy of native PPM (Figure 3(a)), with an overall reduction in nuclei (Figure 3(b)). Similarly, Alcian blue staining suggested a reduction in native sGAG content (Figure 3(c)) in dPPM (Figure 3(d)). PSR labelling under cross-polarised light in native (inset Figure 3(e)) and dPPM (inset Figure 3(f)) demonstrated reduced collagen staining in dPPM in comparison. The gross organisational structure of collagen in native tissue (Figure 3(e)) under polarised light remained relatively conserved in dPPM (Figure 3(f)). VVG staining for elastin fibres (black) in native (Figure 3(g)) and dPPM (Figure 3(h)) also demonstrated reduction in staining intensity for elastin following decellularisation.

### 3.2. Confocal Imaging and Analysis

**Figure 4:**
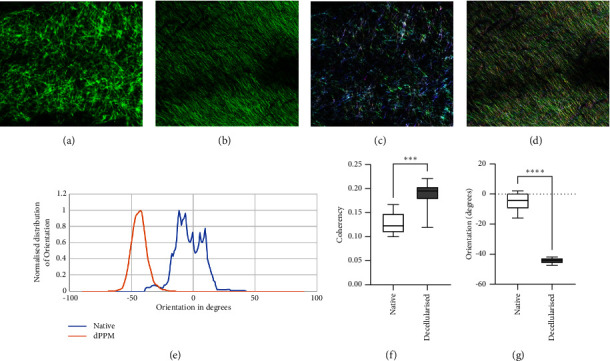
Microstructural alignment of ECM fibres in native and dPPM. Native (a) and dPPM (b) ECM observed under confocal microscopy. Scale = 200 *µ*m. (c, d) Represents colour-coded fibre orientation maps of native (c) and dPPM (d) samples. (e) Represents angular distribution of ECM fibres in native and dPPM. (f) Represents coherency in fibre orientations in native and dPPM. (g) Represents dominant ECM fibre orientations in native and dPPM (*n* = 3, ^*∗∗∗*^*p*  <  0.001, ^*∗∗∗∗*^*p*  <  0.0001, data represented as mean ± SD).

Microstructural changes in the fibrous organisation of native ECM after decellularisation were quantified using the OrientationJ plugin of ImageJ. Images of fresh native (Figure 4(a)) and dPPM (Figure 4(b)) captured with confocal microscopy, were analysed for orientation and coherency. Colour-coded orientation maps generated for native (Figure 4(c)) and dPPM (Figure 4(d)) highlighted predominant orientations of fibres for each, which on further analysis generated a distinct peak for the distribution of orientations of dPPM fibres in contrast with a plateau-like multimodal distribution of fibre orientations in native (Figure 4(e)). Quantitative measurements for orientation also indicated a significant increase in coherency of fibre orientations for dPPM (Figure 4(f)) against native (^*∗∗∗*^*p* < 0.001). Degree of orientation (Figure 4(g)) was in concurrence with Figure 4(e), where native fibres demonstrated a predominant angular position of −5° in contrast with the dPPM fibres assuming an alignment at −44° from the horizontal (^*∗∗∗∗*^*p* < 0.0001).

### 3.3. Bioquantitative Assays

**Figure 5:**
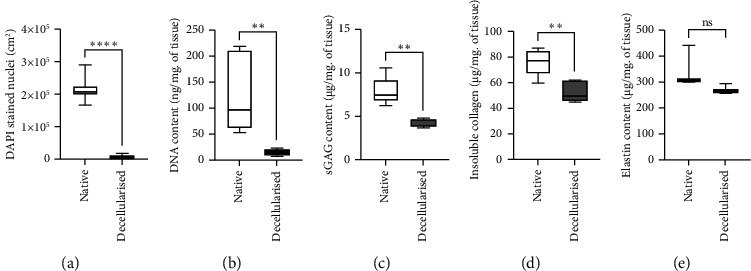
Protocol efficiency in PPM decellularisation. (a) DAPI stained nuclei in native vs. dPPM. (b) DNA content in native vs. dPPM. (c) sGAG content in native vs. dPPM. (d) Insoluble collagen in native vs. dPPM. (e) Elastin content in native vs. dPPM (*n* = 5 biological replicates, ^*∗∗*^*p*  <  0.01, ^*∗∗∗∗*^*p* ≤ 0.0001, data represented as mean ± SD).

Quantification of DAPI-stained nuclei (Figure 5(a)) in native (2.1 × 10^5^ ± 0.3 × 10^5^ nuclei/cm^2^) and dPPM (0.05 × 10^5^ ± 0.06 × 10^5^ nuclei/cm^2^) indicated a 40-fold reduction in dPPM vs. native (*n* = 5, ^*∗∗∗∗*^*p* < 0.0001). This was reflected in the overall DNA content (Figure 5(b)) in dPPM (15.2 ± 5.9 ng/mg. of tissue) versus native tissue (123.5 ± 74.6 ng/mg. of tissue), demonstrating a significant reduction of over 85% of native nuclear DNA (*n* = 5, ^*∗∗*^*p* < 0.01). sGAG quantification (Figure 5(c)) of dPPM (4.2 ± 0.4 *µ*g/mg. of tissue) versus native (7.9 ± 1.6 *µ*g/mg. of tissue) revealed a 53.5% reduction in content (*n* = 5, ^*∗∗*^*p* < 0.01). Insoluble collagen content (Figure 5(d)) was reduced in dPPM (52.7 ± 8.4 *µ*g/mg of tissue) versus native control (76.0 ± 10.5 *µ*g/mg of tissue) with an overall 31% reduction (*n* = 5, ^*∗∗*^*p* < 0.01). In contrast with reduced elastin staining observed in dPPM histology, Fastin quantitative assay demonstrated comparable elastin content (Figure 5(e)) preserved in the decellularised pleura against native control.

### 3.4. Sterility Assessment

**Figure 6:**
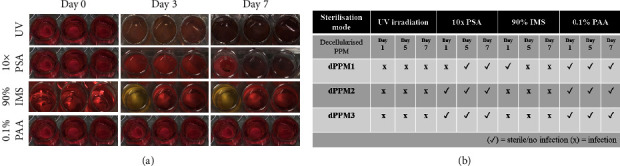
Sterility assessment of dPPM. (a) Sterilisation efficiency of UV, 10x PSA, 90% IMS, and 0.1% PAA at day 0–day 7; (b) dPPM sterility logs from day 0–day 7 (*n* = 3 for each treatment).

UV and 10x PSA-treated samples displayed turbidity and colour change in culture media, in all wells by day 3 of incubation. Similar observations were made with IMS-treated samples with 33% of the wells showing infection by day 3 (Figure 6(a)). 0.1% PAA-treated samples demonstrated no change to medium opacity and colour throughout the incubation period (Figures 6(a) and 6(b)).

### 3.5. Mechanical Characterisation

**Figure 7:**
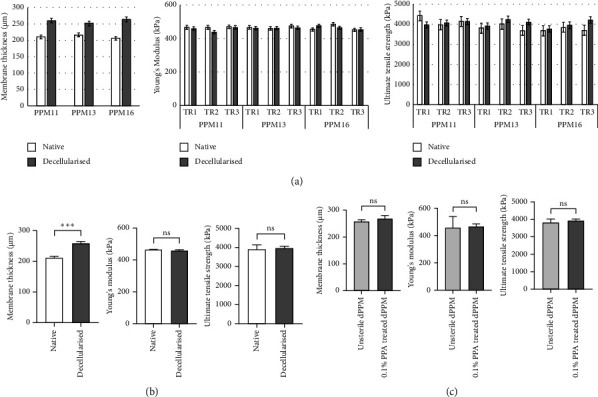
Mechanical characterisation of dPPM against native PPM. (a, b) Estimation of membrane thickness, Young's modulus, and ultimate tensile strength in dPPM against native control. (c) Comparable mechanical properties in 0.1% PAA sterilised dPPM against untreated dPPM (*n* = 3 biological replicates, TR = technical replicate for each PPM sample, ^*∗∗∗*^*p* < 0.001, data represented as mean ± SD).

dPPM displayed an 22.28% increase in membrane thickness (257.92 ± 6.6 *μ*m) against native (210.92 ± 5.34 *μ*m) (*n* = 3 biological replicates, ^*∗∗∗*^*p* < 0.001, data represented as mean ± SD) as seen in Figures 7(a) and 7(b). Stress-strain curves of dPPM under uniaxial tension demonstrated viscoelastic characteristics with an initial toe region, followed by linear and failure regions of response. Core mechanical characteristics of native PPM with an estimated Young's modulus of 465.82 ± 10.51 kPa and ultimate tensile strength of 3912.89 ± 247.42 kPa were conserved in the dPPM, reflected by the comparable values of Young's modulus and ultimate tensile strength of 459.67 ± 10.36 kPa and 4036.22 ± 155.1 kPa, respectively (Figures 7(a) and 7(b)). 0.1% PAA-sterilised dPPM retained comparable mechanical strength and behaviour as observed in untreated dPPM with an estimated Young's modulus of 459.30 ± 79.06 kPa and ultimate tensile strength of 3928.44 ± 85.92 kPa (Figures 7(a) and 7(b)). (*n* = 3 biological replicates, ^*∗∗∗*^*p* < 0.001, ns = nonsignificant, data represented as mean ± SD).

### 3.6. Contact Cytotoxicity Assay

**Figure 8:**
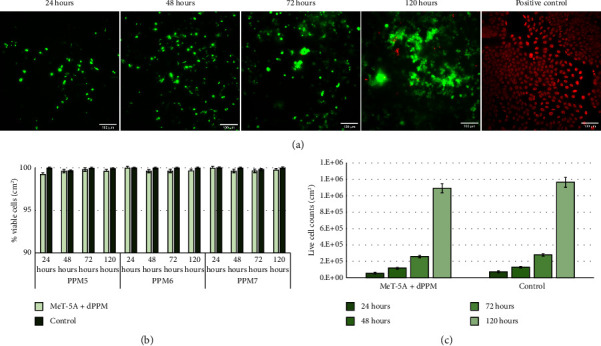
Biocompatibility of decellularised PPM with contact cytotoxicity assay. (a) Live/dead staining of seeded MeT-5A cells in contact with sterilised dPPM on day 1 through day 5. (b) Percentage viability of MeT-5A cells in contact with per sq.cm of dPPM from day 0 through day 5. (c) Live cell counts/cm^2^ of MeT-5A in contact with dPPM against control, on day 1 through day 5 (*n* = 3 biological replicates, data represented as mean ± SD).

Live/dead staining of MeT-5A cells in contact with sterilised dPPM at 24 hours, 48 hours, and on days 3 and 5 demonstrated negligible presence of EthD-1-stained dead cells (Figure 8(a)). The Trypan Blue exclusion assay demonstrated consistent viability (>99%) of MeT-5A in contact with dPPM, from day 1 to day 5 (Figure 8(b)). Comparable viable cell counts of MeT-5A in contact with per sq.cm of dPPM against control, at respective time points confirmed negligible cytotoxicity of dPPM (Figure 8(c)).

### 3.7. Scaffold Biocompatibility

**Figure 9:**
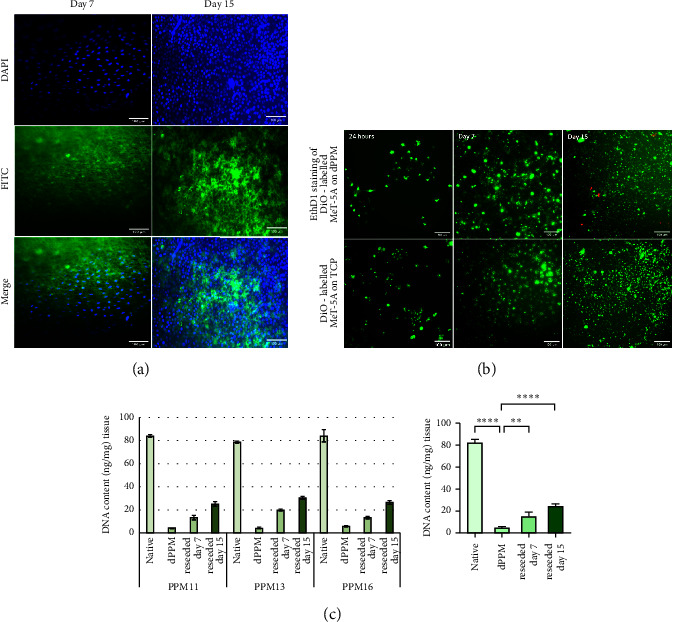
Recellularisation of sterilised dPPM using lipophilic cell tracking dye, DiO-labelled MeT-5A. (a) DAPI staining of reseeded dPPM with labelled MeT-5A on day 7 and day 15. (b) Ethidium homodimer (EthD-1) staining of labelled MeT-5A reseeded on dPPM at 24 hours, day 7 and day 15, against labelled MeT-5A seeded on TCP (control). (c) DNA quantification of reseeded dPPM versus native and unseeded dPPM (*n* = 3, ^*∗∗*^*p*  <  0.01, ^*∗∗∗∗*^*p* < 0.0001, data represented as mean ± SD).

Reseeded dPPM were aseptically transferred to fresh wells for imaging at given time points, to confirm cell adherence to dPPM scaffold and not the tissue culture plate. Nuclear staining with DAPI localised the adherence and proliferation of DiO labelled MeT-5A cells seeded on dPPM, as seen in Figure 9(a) on day 7 and day 15. Consistent viability of seeded MeT-5A was demonstrated with negligible staining of dead cells with viability indicator, Ethidium homodimer (Figure 9(b)). Lipophilic carbocyanine dye, DiO, used to label MeT-5A, permeates the cell membrane and laterally diffuses to stain the entire cell surface. With proliferation of cells in long-term culture, the dye is diluted over time, with labelled membranes internalised within intracellular organelles [35], rendering cells a granular appearance, evident in both reseeded dPPM and TCP control (Figure 9(b)). DNA quantification of reseeded dPPM at day 7 (^*∗∗*^*p* < 0.01) and day 15 (^*∗∗∗∗*^*p* < 0.0001) demonstrated a significant increase in DNA content against unseeded dPPM, indicative of MeT-5A proliferation seeded within the dPPM scaffold (Figure 9(c)).

## 4. Discussion

Pioneered by Meezan et al. in 1975 [36], whole organ and membrane decellularisation have gained a strong foothold in scaffold fabrication in tissue engineering. Preservation of tissue-specific microarchitecture, biochemical composition, and mechanical attributes of native ECM with a reduced risk of immunogenicity form the hallmarks of an optimal decellularisation process. Porcine biological scaffolds as human-scale substitutes, owing to the evolutionarily conserved and comparable ECM characteristics, have found clinical applicability in cardiovascular, [37–39] cranial, [40, 41], and bladder [42–44] reconstruction.

Even with advanced minimally invasive surgical techniques and intraoperative wound closure measures, approximately 5%–10% of patients suffer from postsurgical PAL lasting longer than 7 days, often requiring surgical intervention [21, 45–47]. Kanzaki et al. [48] in 2008 described a TE alternative for functional closure of compromised pleura using transplantable autologous dermal fibroblast cell sheets. Irrespective of outcome, time-bound logistics in deriving and preserving viable autologous cell-sheets, transportation, and storage were potential translation barriers in the clinical application.

Although considerable studies support wound closure techniques that include surgical stapling [49], pleurodesis [50], and application of biological and synthetic sealants [51], the reported scattered efficacy of these techniques in resolving PAL along with the severity of associated complications seen in spontaneous and traumatic pneumothoraces still pose a significant challenge in air leak management. We hypothesised the potential of decellularised porcine pleura as site-specific mechanical barriers or adjuncts, to reinforce current closure techniques in reducing the incidence and severity of PAL.

Studies have adopted various physical, chemical, enzymatic, and combinative methods to optimise decellularisation, specific to the source and nature of the tissue, cellularity, ECM characteristics and intended clinical application [52–55]. The two-step decellularisation approach in our study with physical freeze-thaw cycles followed by treatment with a mild detergent-nuclease buffer was in efforts to globally preserve the structural and biochemical characteristics of native pleura. Intracellular ice crystal formation during freeze-thaw cycles induced cell lysis and subsequent removal of cell debris with periodic washes [55]. Tissues including peripheral nerve grafts, heart valve leaflets, and dermis have been decellularised with this technique, characterised by minimal disruption in gross ECM morphology. However, considerable retention of nuclear material was reported with this process alone [56–58]. Subsequent treatment with a buffer containing low concentrations of Triton-X 100 and sodium deoxycholate (SD) enhanced solubilisation of the cytoplasmic cellular and nuclear content. A milder surfactant, SD has shown enhanced preservation of gross microarchitecture and core composition of native ECM in soft tissue and basement membrane decellularisation [7, 59]. Agglutination of DNA on the tissue surface, often an issue with use of SD was addressed with addition of DNase I in the buffer [7, 60]. The enzyme-detergent extraction system ensured adequate removal of nuclear debris. Use of a recombinant nuclease in the protocol, also reduced the risk of prion disease transmission seen with bovine-sourced enzymes [54].

Periodic washes with PBS and deionised water enhanced the removal of cellular debris and detergent remnants. There is always a certain degree of ECM damage associated with any decellularisation process. In addition to the physical, chemical, or enzymatic disruptive methods that affect ECM composition and organisation, factors like spontaneous tissue breakdown and the release of intracellular proteases during decellularisation, unintentionally cause more damage [7, 61]. From a protocol optimisation perspective, the use of mild denaturing and processing conditions at 4°C at a neutral pH range of 7-8, for most of the duration, helped mitigate the impact of the decellularisation process on native pleural ECM.

Residual DNA in dPPM was less than 50 ng/mg of tissue, with a visible reduction in nuclei, reflecting protocol adherence to the criteria laid down by Crapo and Gilbert [7], for efficient decellularisation. Commercially available decellularised matrices derived from porcine SIS [43], porcine urinary bladder [62], and bovine dermis [63] bearing trace amounts of residual DNA have sought applications in soft tissue reinforcement. Studies have looked at significant reduction in cellularity over complete cell removal as a balanced approach in tailoring tissue-specific decellularisation protocols [7, 54, 55]. This is mainly in consideration of preserving the core ECM structure and physiology for intended clinical applications.

Pleural ECM rich in collagen and elastin, confer viscoelastic characteristics, required in limiting lung distensibility during deep inspiration and generating elastic recoil pressure under resting conditions [64]. Ultrastructural studies attribute the distinct organisational pattern of collagen and elastin fibres in an irregular interwoven plaited structure to be responsible for the mechanical resilience of lungs during respiration [65]. The orientation and composition of the collagenous fibrous matrix equips the pleura to the physiological demands of flexibility along a multidirectional axis, a characteristic to lung volume changes during breathing [28, 30].

The choice of a mild combinative enriching process was to ensure maintaining global structure and compositional integrity of pleural ECM, relevant in the use of dPPM as functional bioscaffolds. dPPM characterisation; however, reflected a degree of compromise to the native biochemical composition and microstructural integrity. Collagen histology showed decreased staining intensity, consistent with a 31% reduction in insoluble collagen content quantified in dPPM. Interestingly, core elastin in pleural ECM that histologically reflected decreased staining, was quantified to be comparable against native control. Previously reported to be considerably labile to SDS/CHAPS [15, 66, 67] and higher concentrations of SDC and Triton-X 100 treatment, [68] retention in native elastin merits the milder protocol used herein.

Microstructural changes to the irregular fibrous meshwork of native pleural ECM were also evident following decellularisation. The coherency in the modified angular distribution of fibre orientation observed in dPPM may be a cumulative result of reduced insoluble collagen density with possible conformational changes in the core protein structure at a molecular level due to denaturing effects of the decellularising agents used [55, 69, 70]. This opens dialogue for comparing the soluble collagen content in dPPM against native control to further characterise the derived scaffolds and potentially explain the reduced insoluble collagen and microstructural realignment in the native ECM following decellularisation. More so, as there is evidence associating fibre alignment and anisotropy with mechanical strength of bioscaffolds [71, 72] and functional behaviour therein.

Several studies have highlighted ultrastructural changes in ECM architecture using scanning electron microscopy and second harmonic generation [70, 73–75]. However, the conflicting degrees of ECM disruption for the same process in different tissues [7, 76, 77] make it difficult to pinpoint the mechanism causing damage at varying levels during the decellularisation process. Research efforts by Hwang et al. to trace spatial and temporal damage to collagen structure at a molecular level with the use of hybridising peptides [69], could be a solution in optimising tissue-specific decellularisation strategies, based on the molecular assessments of structural ECM damage with routinely used decellularising agents like SDS, Triton-X, or SD.

Increased membrane thickness and observed changes in ECM microarchitecture, did not severely alter the mechanical integrity of dPPM [78]. dPPM under uniaxial tension exhibited similar trends in viscoelastic deformation with linear and failure regions of response. This was reflected in the comparable Young's modulus and ultimate tensile strength, indicative of preserved core ECM stiffness and strength, respectively. Native membrane stiffness retained in dPPM is a key physical cue [79] with potential to direct microscale cellular attachment, migration, and subsequent cell-induced tissue remodelling at a macroscale level. The conserved mechanical strength in dPPM could be of interest in exploring orthotopic applications as sealant grafts in the management of PAL and pleural repair.

Pleural ECM, rich in PG and glycoproteins, function as mechanical buffers providing hydration and a repository of bound growth factors that modulate cell behaviour and tissue response to injury and wound healing. sGAG decorating the protein core of PG bind secretory factors including VEGF, EGF, TGF-*β*, and platelet-derived growth factors to be incorporated within the ECM [40, 77, 80]. Significant reduction in sGAG content in dPPM was consistent with findings in other decellularisation studies [77, 79, 81]. Water-soluble sGAG is known to be less resistant to decellularisation, being subsequently lost during repeated wash cycles. Although known for providing intrinsic compressive strength, reports suggest reduction in sGAG do not have an adverse effect on the gross mechanical characteristics of ECM, consistent with the findings herein. From a processing perspective, loss in sGAG improves diffusion of the decellularisation buffer, PBS, and deionised water during subsequent washes, enhancing removal of cellular debris [58]. Additional processing of xenogeneic ECM is important in ensuring safety, biocompatibility, and the “off-the-shelf” potential in clinical applications. The sterilisation study assessing efficiency of several known agents, demonstrated 0.1% peracetic acid to be most effective for dPPM. Routinely used as a mild decellularising agent with low cytotoxicity, PAA is known to be less disruptive to the mechanical and biochemical integrity of the ECM [82, 83].

To maintain tissue relevance, human pleural mesothelial cell line (MeT-5A) [84] was used to assess biocompatibility of dPPM in sustaining cell attachment, viability, and proliferation. Live-dead staining of cells in contact with dPPM at progressive time points in culture demonstrated negligible cytotoxicity. Labelled MeT-5A cellsreseeded on sterile dPPM exhibited comparable adhesion, viability and proliferation as seen with routine culture of labelled MeT-5A on standard tissue culture plastic used as control. Preliminary studies reflecting dPPM cytocompatibility confirms minimal retention of decellularising and processing agents (detergents, nucleases, and sterilant) in the matrix, highlighting protocol safety and efficiency. Although decellularised porcine pleura is relatively unexplored, the study draws parallels with approved bioprosthetic meshes such as urinary bladder matrix (MatriStem®) [85, 86] and mesothelial grafts (Meso Biomatrix®) [87, 88] seeking clinical evaluation in wound healing and soft tissue reconstruction.

## 5. Conclusion

The combinative methodology for decellularising porcine pleura ensured adequate cell removal while preserving global compositional and mechanical integrity of native pleural ECM. Preliminary characterisation reflected dPPM biocompatibility with progressive cell attachment, viability, and proliferation. With dPPM demonstrating inherent mechanical strength and membrane stiffness, the study lays the foundation for future work focusing on site-specific applications of decellularised pleura in air leak management.

## Data Availability

The data used to support the findings of this study are available from the corresponding author upon reasonable request.

## Abbreviations

DAPI: 4′, 6′-diamidino-2-phenyl indole (DAPI)
DMEM: Dulbecco's modified eagle medium
DMMB: 1, 9-dimethyl methylene blue
dPPM: Decellularised pleural membrane
ECM: Extracellular matrix
EGF: Epidermal growth factor
FBS: Fetal bovine serum
FGF: Fibroblast growth factor
H&E: Haematoxylin and Eosin
IMS: Industrial methylated spirits
PAA: Peracetic acid
PAL: Persistent air leaks
PBS: Phosphate-buffered saline
PG: Proteoglycan
PPM: Porcine pleural membrane
PSA: Penicillin/streptomycin/amphotericin B
PSR: Picrosirius red
ROI: Region of interest
SD: Sodium deoxycholate
sGAG: Sulphated glycosaminoglycans
SIS: Small intestinal submucosa
TCP: Tissue culture plastic
TE: Tissue engineering
TGF-*β*: Transforming growth factor-beta
UBM: Urinary bladder matrix
VEGF: Vascular endothelial growth factor
VVG: Verhoeff–Van Gieson
UV: Ultraviolet light.

## Additional Points

*Impact Statement*. Design and development of “off the shelf” tissue-engineered products can rely on decellularisation of native tissue that can be functionalised with cell ingress from surrounding host microenvironment. We describe a reproducible decellularisation method for porcine pleural membranes. Histology, mechanical testing, and biocompatibility studies demonstrated protocol efficiency in adequate removal of native tissue cellularity and retention of gross microarchitecture and bioactivity in the decellularised pleura. The study represents a step forward in establishing an off the shelf potential of decellularised pleura as site-specific mechanical barriers in curtailing prolonged air leaks and promoting spontaneous tissue regeneration with relevant physiological cues.
